# Editorial: Context-Dependent Plasticity in Social Species: Feedback Loops Between Individual and Social Environment

**DOI:** 10.3389/fpsyg.2021.645191

**Published:** 2021-02-01

**Authors:** Mathieu Lihoreau, Sylvia Kaiser, Briseida Resende, Heiko G. Rödel, Nicolas Châline

**Affiliations:** ^1^Research Center on Animal Cognition (CRCA), Center for Integrative Biology (CBI), CNRS, University Paul Sabatier-Toulouse III, Toulouse, France; ^2^Department of Behavioural Biology, University of Münster, Münster, Germany; ^3^LEDIS, Department of Experimental Psychology, Instituto de Psicologia, University of São Paulo, São Paulo, Brazil; ^4^Programa de pós-graduação em Psicologia Experimental, University of São Paulo, São Paulo, Brazil; ^5^Laboratoire d'Ethologie Expérimentale et Comparée UR 4443, Université Sorbonne Paris Nord, Villetaneuse, France; ^6^LEEEIS, Department of Experimental Psychology, Instituto de Psicologia, University of São Paulo, São Paulo, Brazil

**Keywords:** social behavior, ethology and behavioral ecology, interactions and social networks, social stress, sociality and complexity, coping style, social umwelt, social niche construction

## Introduction

Behavioral sciences cover a wide range of research fields, such as ethology, behavioral ecology, behavioral genetics, and behavioral physiology. In all these fields, individuality in behavior is now widely recognized and thus the focus of many studies is shifting from population averages to individual differences (Wolf and Weissing, 2012). Studies of inter-individual behavioral differences, although descriptive and functional in the beginning (Sih et al., 2004), start once again to address the four fundamental questions of ethology (Tinbergen, 1963), and to focus on developmental and mechanistic aspects (Jeanson and Weidenmüller, 2014). Some attempts also integrate both, inter and intra-individual variations in a more general framework of research (Dingemanse et al., 2010). Given this renewed interest in understanding whether or not changes in individual behavioral decisions occur, it is more important than ever to elucidate how plasticity (developmental, contextual, etc.) combines with non-plastic inter-individual variation, and how norms of individual reaction are essential to elicit the diverse individual behaviors in different contexts.

Social interactions constitute a significant part of an individual's experience (Hinde, 1976; Sachser et al., 2020). As such, these interactions are both immediate contextual factors and selective pressures on the expression of adapted behavioral responses. Behavioral decisions greatly depend on what can be considered a “social umwelt” (von Uexküll, 1921; Yamagishi and Hashimoto, 2016), which is both part of the perceptual environment as well as a way of perceiving the environment, allowing individuals to act upon a diversity of cues and signals. This potential richness in contextual variation has certainly shaped the individual behavior of social species, having consequences on decision mechanisms, such as the cognitive mechanisms associated with social life.

Recent studies have shown how social context and experience can change behavioral decisions of a variety of social species (Yagound et al., 2012; Fragaszy et al., 2017). Ultimately, the social environment can even create such an intense stress response that animals have difficulties showing sufficient plasticity or adaptive responses, a current and relevant problem in a period of accelerated ecological changes (Harlow et al., 1965; Koolhaas et al., 2017; Takahashi et al., 2018; Balasubramaniam et al., 2020). This new focus on how plasticity and inter-individual differences influence social behavior makes it timely to join different perspectives and aggregate new findings of various fields. Experimental evidence of the context-dependent plasticity from diverse organisms can lead to the elaboration of a common research framework, bringing back comparative psychology and ethology in the understanding of social behavior, its expression, development, ecology and evolution in an overt fashion.

In this Research Topic, we addressed the mechanisms behind inter and intra-individual variation and/or consistency in behavioral expression focusing on social interactions. Our goal was to explore how experience affects all levels of behavioral complexity, from molecular to population-level approaches.

## The Social Environment Shapes Behavioral Profiles

Social interactions profoundly influence behavioral development and expression. For instance, it has long been shown that social isolation during development can induce behavioral disturbances in group-living species (e.g., primates: Harlow et al., 1965; insects: Lihoreau et al., 2009). Here several mechanisms by which the social environment influences behavioral flexibility are highlighted.

Behavioral flexibility can depend on group composition. Hemelrijk et al. analyzed a seven years dataset of wild vervet monkeys (*Chlorocebus pygerythrus*) and found that female dominance to males varies relative to the number of males in the group: the more males, the higher the proportion of fights won by females. In this species, intersexual dominance presumably emerges through a winner-loser effect whereby winners of an aggressive interaction tend to win more and more (Dugatkin, 1997). Mutwill et al. showed that behavioral flexibility can also depend on social status. Domestic guinea pigs (*Cavia aperea* f. *porcellus)* live in large mixed-sex groups with a dominance and queuing system that gives older males an advantage for accessing females. By conducting paternity analyses on different colonies, Mutwill et al. demonstrated that both young and old males can nevertheless attain comparable reproductive success. Thus, younger males reproduced irrespective of queuing and their low social status. Males may use different reproductive tactics (sneaking, fighting, bonding with a specific female), which are flexibly applied with the onset of sexual maturity. Kraus et al. performed cross-fostering experiments in guinea pigs of wild origin demonstrating that behavioral and physiological differences between pups were plastic. This study illustrates the combined influence of the social environment and pre and post-natal experience as determinants of adaptive flexibility.

Interestingly however, these effects of the social environment are not expressed in every context. Yoshida and Koda investigated the behavior of goats (*Capra hircus*) facing an unsolvable task (i.e., in which food reward was kept in a sealed transparent bucket in the presence of a human), and observed that the social rank of the goats did not influence their behavior. In this context, inter-individual differences in sociability toward humans best predicted the behavioral responses, as only the most sociable goats sleeked help from humans to solve the task.

## From Inter-Individual to Inter-Group Differences

Inter-individual behavioral variance can also shape the social environment and the behavior of groups. This is, for instance, the case in colonies of social insects that rely on division of labor between physiologically, morphologically and behaviorally distinct classes of individuals (Hölldobler and Wilson, 2009). Recent studies put forward the critical importance of behavioral heterogeneity for collective behavior (Jolles et al., 2020).

Kolay et al. reviewed growing evidence of personality within castes of ants. Foragers, in particular, can show persistent inter-individual variability in their incentive to start foraging after receiving food, deposit trail pheromone, be aggressive, be attracted by light, respond to sucrose, or learn. Many of these traits likely influence how individuals perceive and use information, prioritize personal or social information, and learn, which may influence task specialization. The distribution of personalities in a colony can ultimately determine variability in group behavior, for instance with some colonies that are consistently more aggressive than others (Pinter-Wollman, 2012). Japyassu et al. discuss whether the detection of repeated group differences across a population deserves to be considered as a “social personality.” Using an epistemological approach, they argue that socially self-organized systems, such as isolated ant trails and bee recruitment groups, are too simple to have personalities. They advocate social personality should be used as a metaphor rather than a real transposition of a psychological phenomenon, highlighting the great care that should be taken when trying to apply concepts derived from human psychology to non-human cognition (Baracchi et al., 2017). This limitation of the cross-talking between psychology and ethology is also exemplified by Oberhauser et al. who explored cognitive biases in value perception by ant colonies. In humans, expectations are a strong driver of perceived value causing an undervaluation of a given option if a better option was expected, and an overvaluation if a poorer one was expected (e.g., Jayles et al., 2017). Oberhauser et al. tested whether the presence of a pheromone trail influenced the perceived value of a food source in foragers of the black garden ant (*Lasius niger*) navigating a Y-maze. Their results clearly show that trail pheromone, a source of social information, does not distort the value of food sources in these ants.

## Perspectives

Contributions in this Topic Research illustrate the importance of the social environment in shaping behavior and social interactions across taxa (e.g., insects, rodents, ungulates, primates) and contexts (e.g., mating, dominance, collective decisions). However, it also questions about the relevance and care that needs to be taken when applying psychological concepts to non-human animals, and changing scales of observations. We believe that much progress into research on the causes and consequences of behavioral flexibility will greatly benefit from recent technological advances to record and analyse behavior from large numbers of individuals and over long periods of times (Châline et al., 2017; Brown and de Bivort, 2018; Marchal et al., 2020).

## Author Contributions

All authors listed have made a substantial, direct and intellectual contribution to the work, and approved it for publication.

## Conflict of Interest

The authors declare that the research was conducted in the absence of any commercial or financial relationships that could be construed as a potential conflict of interest.
